# Transition metal complexes of phyllobilins – a new realm of bioinorganic chemistry

**DOI:** 10.1039/c5dt00474h

**Published:** 2015-04-29

**Authors:** Chengjie Li, Bernhard Kräutler

**Affiliations:** a Institute of Organic Chemistry & Centre of Molecular Biosciences , University of Innsbruck , Innrain 80/82 , A-6020 Innsbruck , Austria . Email: bernhard.kraeutler@uibk.ac.at

## Abstract

Natural cyclic tetrapyrroles feature outstanding capacity for binding transition metal ions, furnishing Nature with the important metallo-porphyrinoid ‘Pigments of Life’, such as heme, chlorophyll (Chl) and vitamin B_12_. In contrast, linear tetrapyrroles are not generally ascribed a biologically relevant ability for metal-binding. Indeed, when heme or Chl are degraded to natural linear tetrapyrroles, their central Fe- or Mg-ions are set free. Some linear tetrapyrroles are, however, effective multi-dentate ligands and their transition metal complexes have remarkable chemical properties. The focus of this short review is centred on such complexes of the linear tetrapyrroles derived from natural Chl-breakdown, called phyllobilins. These natural bilin-type compounds are massively produced in Nature and in highly visible processes. Colourless non-fluorescing Chl-catabolites (NCCs) and the related dioxobilin-type NCCs, which typically accumulate in leaves as ‘final’ products of Chl-breakdown, show low affinity for transition metal-ions. However, NCCs are oxidized in leaves to give less saturated coloured phyllobilins, such as yellow or pink Chl-catabolites (YCCs or PiCCs). YCCs and PiCCs are ligands for various biologically relevant transition metal-ions, such as Zn(ii)-, Ni(ii)- and Cu(ii)-ions. Complexation of Zn(ii)- and Cd(ii)-ions by the effectively tridentate PiCC produces blue metal-complexes that exhibit an intense red fluorescence, thus providing a tool for the sensitive detection of these metal ions. Outlined here are fundamental aspects of structure and metal coordination of phyllobilins, including a comparison with the corresponding properties of bilins. This knowledge may be valuable in the quest of finding possible biological roles of the phyllobilins. Thanks to their capacity for metal-ion coordination, phyllobilins could, *e.g.*, be involved in heavy-metal transport and detoxification, and some of their metal-complexes could act as sensitizers for singlet oxygen or as plant toxins against pathogens.

## Abstract 2

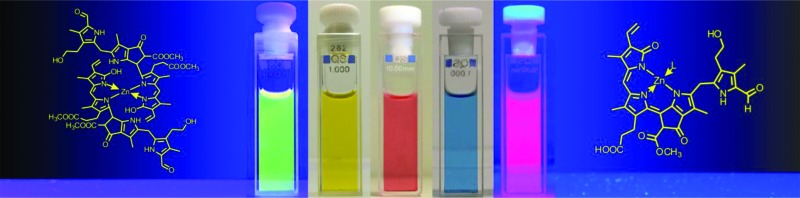
Phyllobilins may function as natural ligand molecules for biologically important transition metal ions, giving complexes with remarkable chemical and photophysical properties.

## Introduction

Cyclic tetrapyrroles feature outstanding capacity for binding (transition) metal ions, furnishing Nature with the important metallo-porphyrinoid ‘Pigments of Life’,^1^ such as heme, chlorophyll and vitamin B_12_.^2–4^ Linear tetrapyrroles, in contrast, are not typically ascribed a biologically relevant ability for metal-binding.^5–9^ Most known natural linear tetrapyrrroles are products of the degradation of heme^5,10–12^ or of chlorophyll,^13^ such as bilirubin (BR)^6^ or, *e.g.*, ‘non-fluorescent’ chlorophyll catabolites (NCCs), respectively.^9,11,14^ When these linear tetrapyrrroles are produced *via* their respective catabolic pathways, the central Fe- and Mg-ions of heme or of chlorophyll are liberated from their porphyrinoid encasement, to be recuperated for the purpose of alternative metabolic uses.^10,12^

The ‘bile-pigments’ (or natural heme-derived bilins) were already studied in ancient times, when they were puzzling, originally, as enigmatic secretions in animals and humans.^5,6^ Nowadays, bile-pigments are ascribed intricate physiological roles (see, *e.g.* ref. 6 and 15–17). In more recent times, attention has also been drawn to heme-derived bilins in plants and in other photosynthetic organisms, where they play important roles, *e.g.*, in absorbing and sensing sunlight.^7,18,19^ Phyllobilins, on the other hand, the natural degradation products of chlorophyll (Chl) in higher plants, are a related type of linear tetrapyrroles,^9,11^ which, surprisingly, has come into our focus only rather recently.^14,20^ Chl-degradation is a natural phenomenon, commonly associated with the seasonal appearance of the fall colours.^21^ Each year, it provides the biosphere the astounding estimated amount of about 10^9^ tons of phyllobilins.^22^ Chl-derived bilin-type compounds are, thus, economically and ecologically relevant, and no less fascinating than other natural bilins.^23^

This ‘Perspective’ deals with phyllobilins, primarily, and compares them with bilins. It focuses on the role of both types of such linear tetrapyrroles as ligands for transition metal-ions, and on the structure and chemical properties of the resulting metal-complexes.^24–26^

## Phyllobilins – a new type of natural bilins

**Fig. 1:**
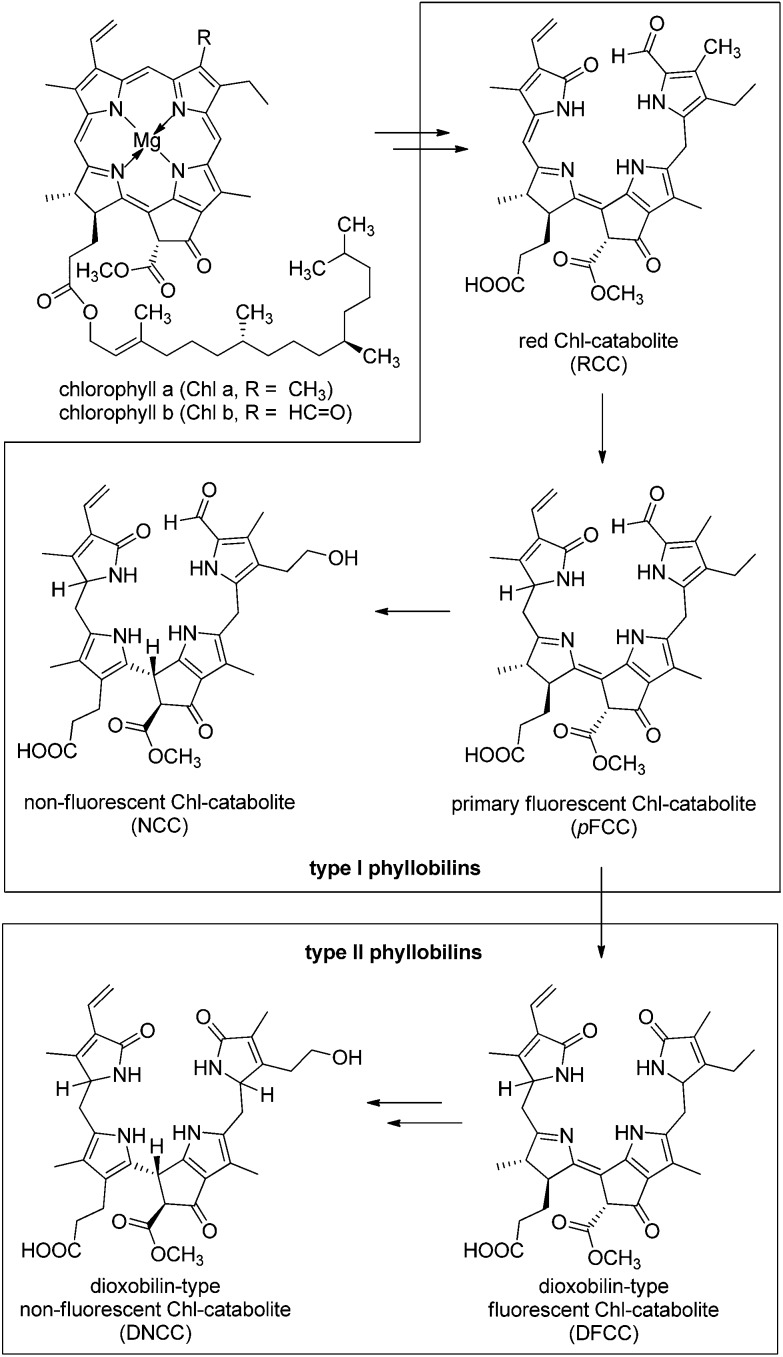
Typical Chl-catabolites (phyllobilins) of the PaO/phyllobilin pathway of Chl-breakdown.^9^ 1-Formyl-19-oxobilins (or type-I phyllobilins) are produced first, by oxygenolytic cleavage of the chlorin macrocycle. In a later step, the formyl group is removed, giving access to 1,19-dioxobilins (or type-II phyllobilins). In typical de-greening leaves, colourless ‘non-fluorescent’ Chl-catabolites (NCCs) and/or dioxobilin-type NCCs (DNCCs) accumulate temporarily as representative type-I or type-II phyllobilins, respectively.

Phyllobilins, the tetrapyrrolic products of the natural degradation of chlorophyll (Chl), are generated in plants at a massive scale.^9^ Chl-breakdown was remarkably enigmatic until about 25 years ago, since - up to then - no trace of Chl-degradation products had been found. In 1991 a non-fluorescent Chl-catabolite (NCC) was described as a 1-formyl-19-oxobilin-type linear tetrapyrrole.^20^ This finding opened the door to the identification of the phyllobilins,^9^ and to the structure-guided discovery of the ‘PaO/phyllobilin’ pathway of Chl-breakdown in higher plants,^13,23,27–29^ which is relevant, both in senescence,^30^ and in fruit ripening.^31–33^ More recently, the PaO/phyllobilin pathway was recognized to ‘branch out’ and to furnish a second type of bilin-type Chl-catabolites.^34,35^ The most widely occurring (colourless) representatives of the latter have been classified as ‘dioxobilin-type’ non-fluorescent Chl-catabolites (DNCCs), which share a common 1,19-dioxobilin-type structure with the now ‘classical’ heme-derived bilins.^5^ DNCCs are formal deformylation products of NCCs, but do actually arise from oxidative enzymatic de-formylation of fluorescent Chl-catabolite (FCC) precursors, catalyzed by a new type of cytochromes P450.^23,36^ The first formed FCC, or ‘primary’ FCC (pFCC), in turn, results from enzymatic reduction of the red Chl-catabolite (RCC).^37–39^ Most FCCs are only fleetingly existent, blue fluorescent^40,41^ intermediates of Chl-breakdown that are generally ‘programmed’^42^ for direct and rapid conversion (*via* acid catalysis) into the corresponding NCCs (see Fig. 1).^9,43,44^

**Fig. 2:**
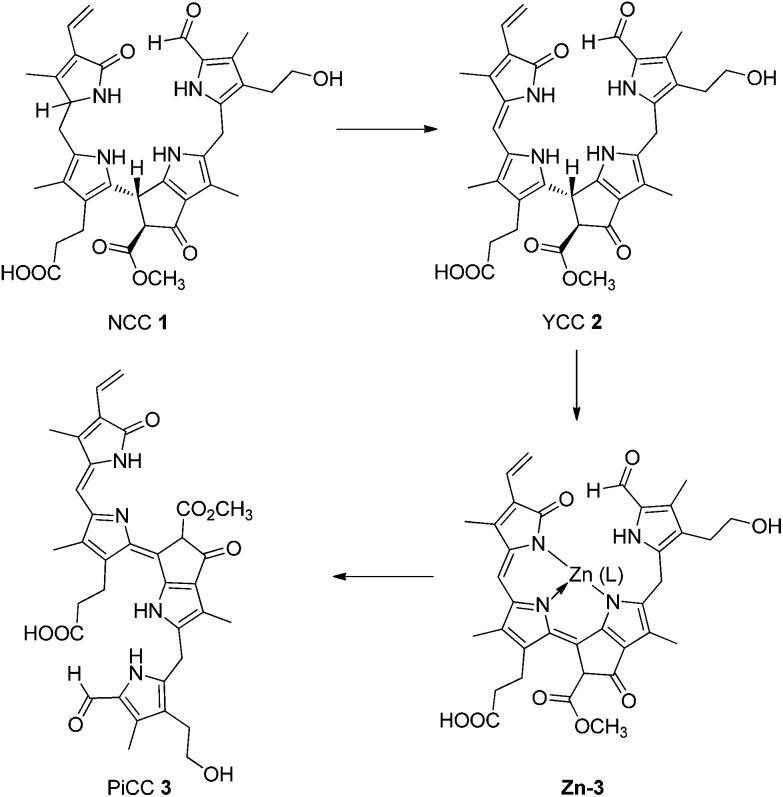
Coloured Chl-catabolites (yellow and pink coloured YCCs and PiCCs) result from ‘biological’ and from ‘chemical’ oxidation of the colourless ‘non-fluorescent’ Chl-catabolites (NCCs): NCC **1** is thus oxidized to the yellow YCC **2**. The latter is further oxidized to the PiCC **3** *via* the blue Zn-complex **Zn-3**.

Recently, yellow Chl-catabolites (YCCs) and pink chlorophyll catabolites (PiCCs) were shown to constitute a new class of natural coloured phyllobilins,^45,46^ which appear to be formed in leaves from NCCs *via* an enzymatic oxidation at later stages of senescence.^47^ The representative YCC **2** was also prepared from the NCC **1** by oxidation with dicyano-dichlorobenzoquinone (DDQ).^45^ YCC **2** was converted into the corresponding PiCC **3** *via* an efficient two-step procedure involving spontaneous air oxidation in the presence of Zn-ions (Fig. 2).^26^

**Fig. 3:**
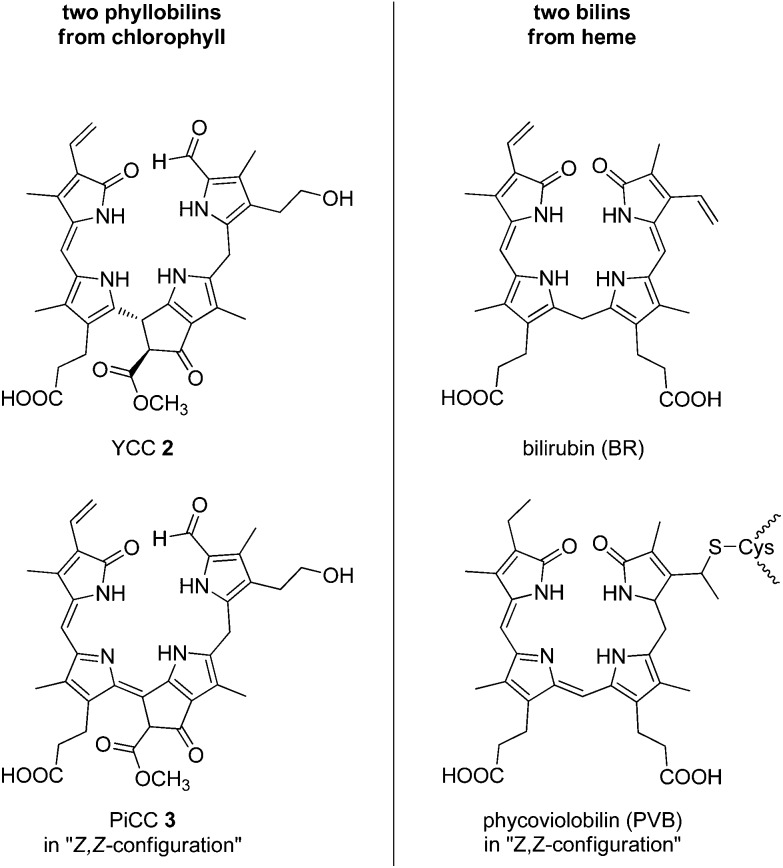
Structural parallels between phyllobilins and heme-derived bilins, as exemplified by the common main chromophores of YCCs (such as **2**) *vs*. bilirubin, and of PiCCs (such as **3**) *vs*. phycoviolobilin.

**Fig. 4:**
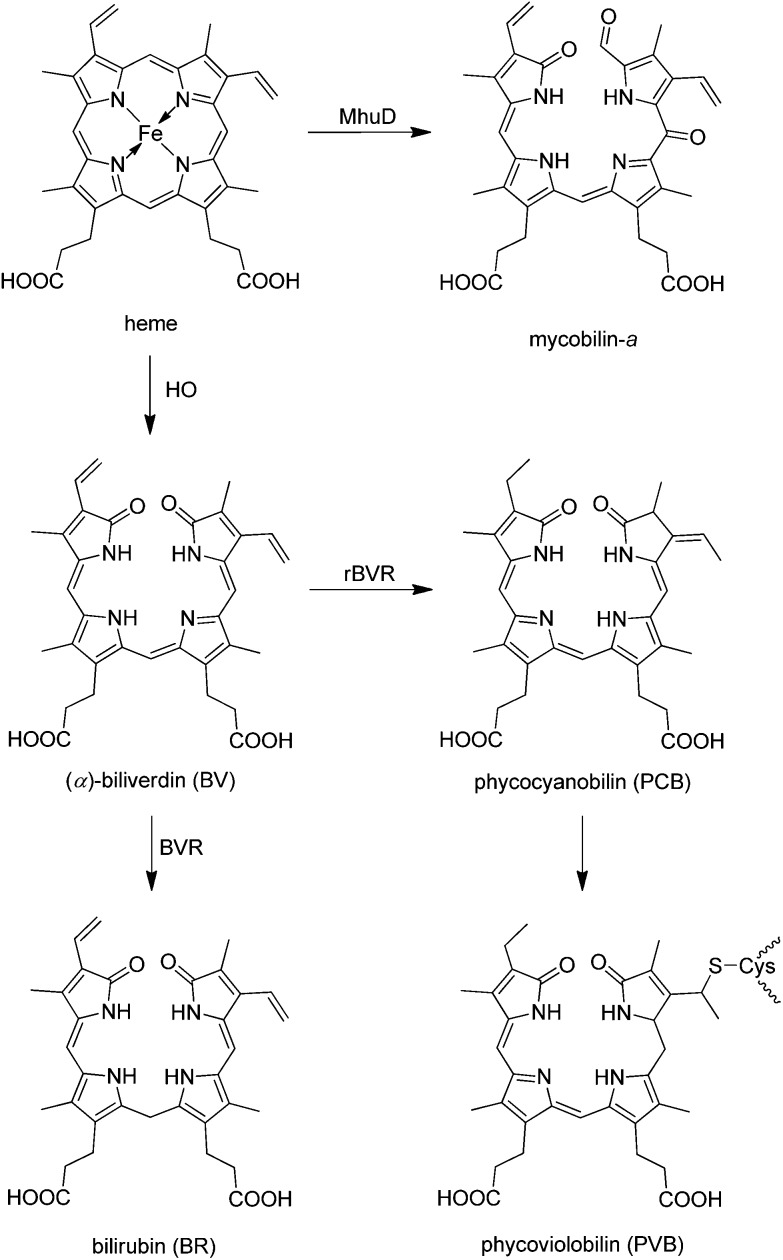
Structures of some natural heme-derived bilins (BVR = BV-reductase, rBVR = radical BVR, HO = heme oxygenase, MhuD = Mycobacterium heme utilization degrader).

At this stage, more than a dozen NCCs^9,32,48^ as well as a range of FCCs^9^ and YCCs^9^ with different structures have all been identified as 1-forml-19-oxo-bilins, now classified as ‘type-I phyllobilins’.^9^ Likewise, several DNCCs^32,34–36,48,49^ and related ‘type-II phyllobilins’^9,49^ have meanwhile been discovered, expanding the repertoire of the known natural phyllobilins, as well as our knowledge on these tetrapyrrolic natural products. Coloured phyllobilins of the type of YCCs and PiCCs have π-conjugated systems extending over two or three pyrrole-type rings, respectively. Remarkably, the main chromophores in YCCs and in bilirubin are virtually identical,^6,50^ and close structural parallels also exist between PiCCs and some natural phycobilins, such as phycoviolobilin, (see Fig. 3 and 4).^7,19,51^

## Bilins – natural linear tetrapyrroles from heme-breakdown

Oxidative cleavage of heme by heme oxygenase (HO) at its (‘northern’) α-*meso* position gives (α)-biliverdin (BV), CO and an Fe-ion,^10^ providing a highly regio-selective entry to the bile pigments.^5^ Subsequent reduction of BV by NADPH, catalyzed by BV-reductase (BVR), generates bilirubin (BR) in animals and in humans.^52^ For the purpose of making BR more water soluble and available for excretion, it is then conjugated with glucuronic acid in the liver.^6^ Phycobilins (*e.g.*, phyco-cyanobilin, phyco-violobilin and phyco-urobilin, see, *e.g.* ref. 8, 18, 19 and 51) form another widespread group of linear tetrapyrroles derived from BV and produced in photosynthetic organisms (plants, bacteria and algae) by enzyme-catalyzed reduction of BV by radical BVRs (*r*BVRs).^7,53^

Alternative natural pathways of heme-degradation that furnish regio-isomers of (α)-biliverdin (BV) have been discovered.^12^ Furthermore, the oxygenase MhuD, a ‘non-canonical’ new type of oxygen-dependent heme-degrading enzyme was recently found in mycobacteria.^54,55^ In contrast to ‘classical’ heme-oxygenase (HO), MhuD converted heme into a 1-formyl-oxobilin (mycobilin) regio-selectively, without generating CO (Fig. 4). Interestingly, these heme-catabolites carry functionalities at the cleavage site that remind of some chlorophyll catabolites, now classified as type-I phyllobilins (see above).

## Man-made linear tetrapyrroles

‘Coupled oxidation’ of heme (or of its dimethyl ester) with ascorbate and oxygen was studied as a model reaction for the oxidation of heme to BV by HO.^24^ It showed insignificant regio-selectivity and the bilin-type products were obtained as a mixture of all four regio-isomers.^56^ The iron complex of (the symmetric) octaethylporphyrin underwent coupled oxidation to give a bilin-type tetrapyrrole with good yield.^57^ The regio-selectivity of ring-opening of heme by HO to (α)-BV is thus explained by directing effects of the protein environment.^55^

**Fig. 5:**
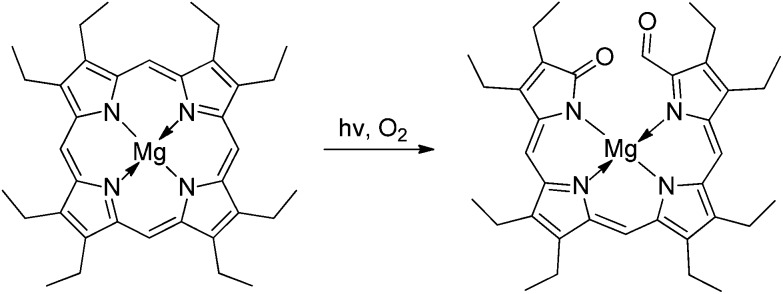
1-Acyl-19-oxobilins are readily available from photo-oxygenation of symmetrical porphyrins, such as Mg-octaethylporphyrin.

Photo-oxygenation reactions of Mg- and Zn-complexes of symmetrical porphyrins (such as octaethyl- and *meso*-tetraphenyl-porphyrin) provided corresponding 1-acyl-19-oxo-bilins from the alternative oxidative cleavage of the porphyrin macrocycle by singlet oxygen, which occurred readily and without loss of the oxidized *meso*-carbon (see Fig. 5).^58–60^

However, when the photo-oxygenation reaction of TPP was done in H_2_O or MeOH, biliviolin analogues were isolated as products of the further addition of water (or methanol) at one of bilitriene's *meso*-positions (see Fig. 15, below).^59,61^

**Fig. 6:**
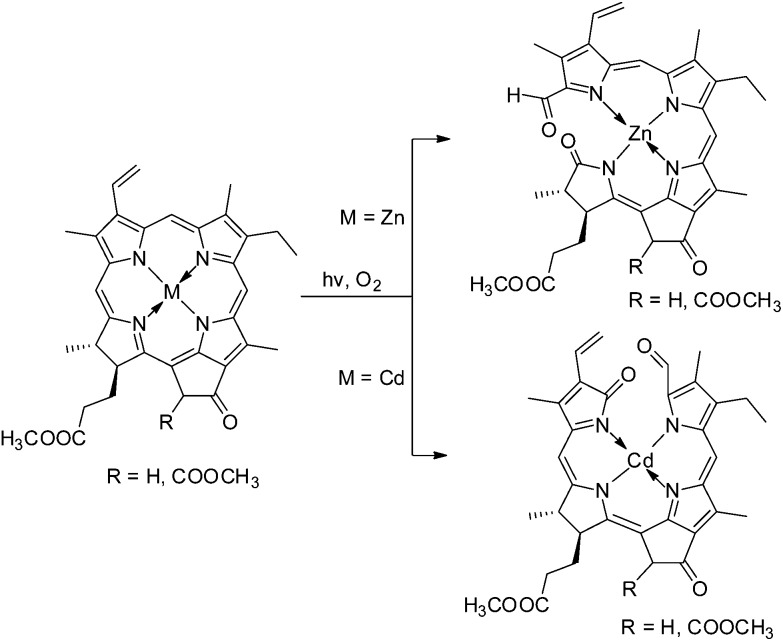
Photo-oxygenolysis of Zn(ii)- and Cd(ii)-(pyro) pheophorbidates allows the efficient preparation of 1-formyl-19-oxobilins with regio-selective cleavage of the macrocycle at the ‘western’ δ-*meso* position or at the ‘northern’ α-*meso* position, respectively.

In the context of the search for synthetic roads to (then still elusive) Chl-catabolites, photo-oxidation of Chl-derivatives was studied as a method for the preparation of formyl-oxo-bilin-type tetrapyrroles.^22,62^ More recently, photo-oxygenation reactions with Zn- or Cd-complexes of methyl pheophorbide *a* ^37^ or of methyl pyropheophorbide *a* or *b* ^63,64^ were found useful and they allowed the partial synthesis of Chl-catabolites found in plants^37,44^ or in a green alga.^65^ Indeed, this method provided 1-formyl-19-oxo-bilins with some regioselectivity, depending upon the coordinated metal ion, with preferential ring opening in the ‘North’ with Cd-complexes, and in the ‘West’ with Zn-complexes (see Fig. 6).^65^

## Transition metal complexes of linear tetrapyyroles

### Transition metal complexes of phyllobilins

**Fig. 7:**
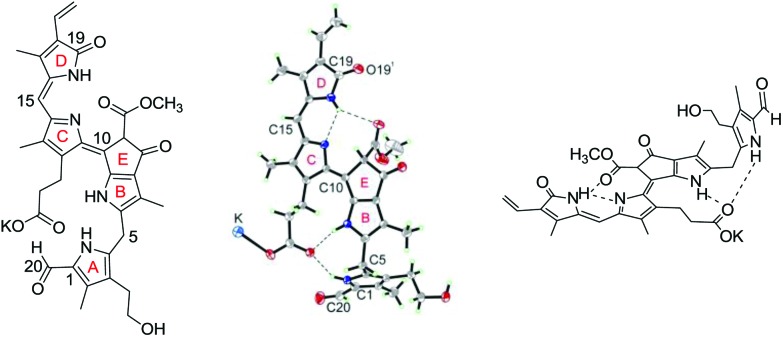
The pink-coloured phyllobilin PiCC **3** exhibits an *E*-configurated C10=C11 double bond, giving it a ‘stretched’ structure in solution and in the crystal. Chemical formula (left), crystal structure (centre) and model structure with highlighted H-bonds (right) are depicted.

Metal complexes of the colourless NCCs are unknown and, indeed, NCCs are not expected to bind transition metal ions,^26^ as deduced for other tetrapyrroles with isolated pyrrole units.^3^ However, NCCs readily oxidize, and more unsaturated phyllobilins are obtained by oxidation of NCCs with DDQ.^45^ By this approach, *e.g.* YCC **2** and PiCC **3** were prepared from the NCC **1**, which feature two or three conjugated pyrrolic rings. YCC **2** and PiCC **3** are natural chlorophyll catabolites that are also found, *e.g.*, in senescent leaves of the Katsura tree (*Cercidiphyllum japonicum*).^46^ The UV-Vis spectrum (in MeOH) of the pink coloured PiCC **3** has strong bands at 313 nm and 523 nm, and solutions of **3** only show a very weak luminescence near 615 nm.^26^ Unexpectedly, the solution structure of PiCC **3** was revealed by NMR analysis with double bonds C10=C11 and C15=C16 with *E*-configuration and *Z*-configuration, respectively.^46^ X-ray analysis of the crystal structure of the potassium salt of **3** (**K-3**) confirmed the NMR-derived structure and revealed bond-lengths consistent with a pattern of single and double bond alternation, as depicted by the formula used (see Fig. 7).^26^ In this first crystal structure of a phyllobilin from a higher plant, **K-3** was revealed to be present as a H-bonded and K-bridged pair of enantiomers, which showed nearly parallel planes of the π-system extending over rings B to D. The three conjugated rings (B, C and D) form a planar structure. The fourth pyrrole (ring A) is stabilized in its ‘out-of-plane’ conformation by an H-bond between the carboxylic acid group and the NH group (of ring A), reminding of the structuring H-bonds observed in the crystal of bilirubin.^6,66^

**Fig. 8:**
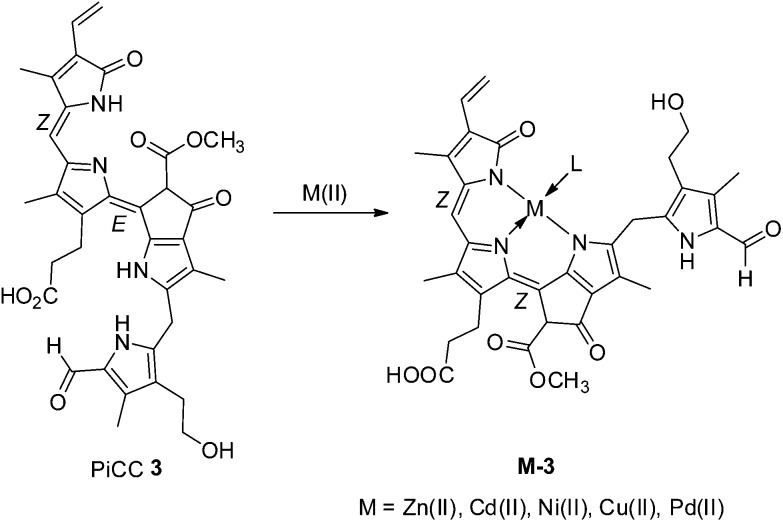
PiCC **3** is effectively tridentate when binding transition metal ions **M(ii)** (such as Zn(ii)-, Cd(ii)-, Ni(ii)-, Cu(ii)- and Pd(ii)-ions).^26,67^ Blue complexes **M-3** are efficiently formed, requiring isomerisation of the C10=C11 double bond of **3** to a *Z*-configuration in **M-3**.

In contrast to NCCs, the coloured phyllobila-*c*,*d*-diene **3** proved to be an excellent multi-dentate ligand for transient metal complexes.^26,67^ Deep blue metal complexes **M-3** (M = Zn, Cd, Ni, Cu, Pd) of PiCC **3** could be prepared in excellent yields by treatment of **3** with corresponding transition metal salts (Fig. 8). Detailed structure analysis of these metal complexes by NMR, suggested a monomeric nature in solution and tridentate coordination of the metal-ion by the ligand nitrogen atoms. Observation of a ring A NH-signal in the ^1^H-NMR spectrum of Zn-PiCC (**Zn-3**) was consistent with this. Polar solvent molecules or the OH group at C3^2^ of ring A are likely fourth ligands (L) at the coordinated metal-ion. In order to achieve a tridentate coordination by PiCC, the metal complexes required a *Z*-configuration of the C10=C11 double bond, not directly compatible with the known, original structure of PiCC (see discussion below). Clearly, phyllobiladienes, such as **3**, represent a new type of natural oligopyrrole that binds transition metal ions very well.^26^

**Fig. 9:**
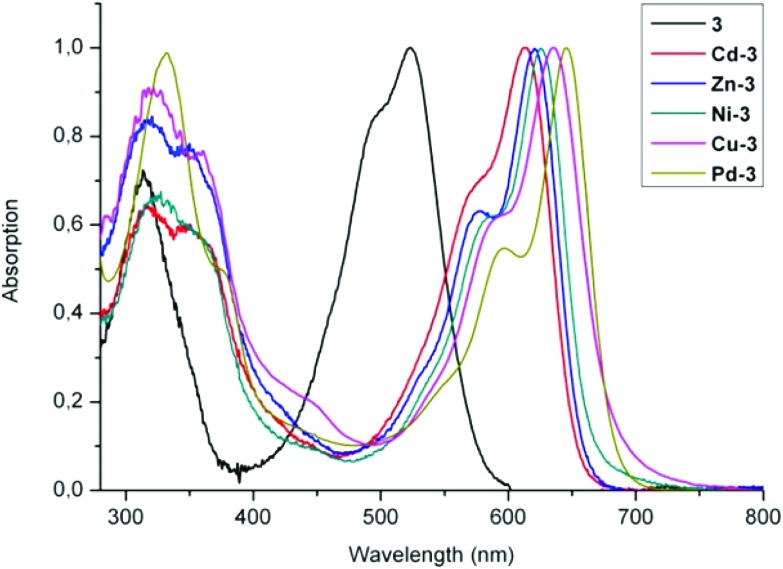
UV-Vis spectra of PiCC **3** (black trace) and of its complexes **M-3** with Cd(ii)-, Zn(ii)-, Ni(ii)-, Cu(ii)- and Pd(ii)-ions, depicted as red, blue, green, violet, and olive-green traces, respectively.

Formation of transition metal complexes (**M-3**) from PiCC **3** was accompanied by colour changes from pink-red to blue,^26^ revealing a notable bathochromic shift of the absorption maximum by roughly 100 nm (Fig. 9). Among the blue complexes **M-3** prepared (with M = Zn, Cd, Ni, Cu and Pd), binding of Pd(ii)-ions to **3** induced the largest bathochromic shift of the absorption maximum (to 645 nm).^67^ Such significant long wavelength shifts can be directly attributed to metal binding in combination with *E* to *Z* isomerization of the C10=C11 double bond. Roughly similar spectral changes were also reported for transition metal-ions, when giving complexes with other linear tetrapyrroles.^68–70^ The capacity of PiCC **3** to bind different transition metal ions (such as Zn(ii)-, Cd(ii)-, Ni(ii)-, Cu(ii)-, and Pd(ii)-ions) reminds of related properties of natural tripyrrolic alkaloids^71^ and of artificial tripyrrones.^25^

The kinetics of formation of several metal-complexes **M-3** of PiCC **3** was analysed qualitatively. At room temperature and in methanol as solvent over-all rates of roughly 600, 200, 10, 400, 1 M^–1^ s^–1^ were determined for Zn(ii)-, Cd(ii)-, Ni(ii)-, Cu(ii)- and Pd(ii)-incorporation from the corresponding metal acetates.^26,67^ Due to the different configuration of the C10=C11 double bond in the ligand **3** and in complexes **M-3** an *E* to *Z* isomerization of the C10=C11 double bond during the complex-formation was inferred.^26^ In the course of the fast formation of **Zn-3**, **Cd-3** and **Cu-3** the deduced double bond isomerization appears to be too fast, to allow the observation of separate intermediate states during complex-formation. However, a first fast interaction of **3** with Ni (indicated by partial spectral changes) in the formation of **Ni-3** is followed by a slow product-forming step, which, presumably, is rate-limited by the isomerization. This result indicated weak bi-dentate coordination of the conjugated C–D-moiety to the metal-ion, as first step in the formation of **M-3**, followed by the double bond isomerization and tridentate coordination of the metal-ion, to afford the stable metal complexes.

**Fig. 10:**
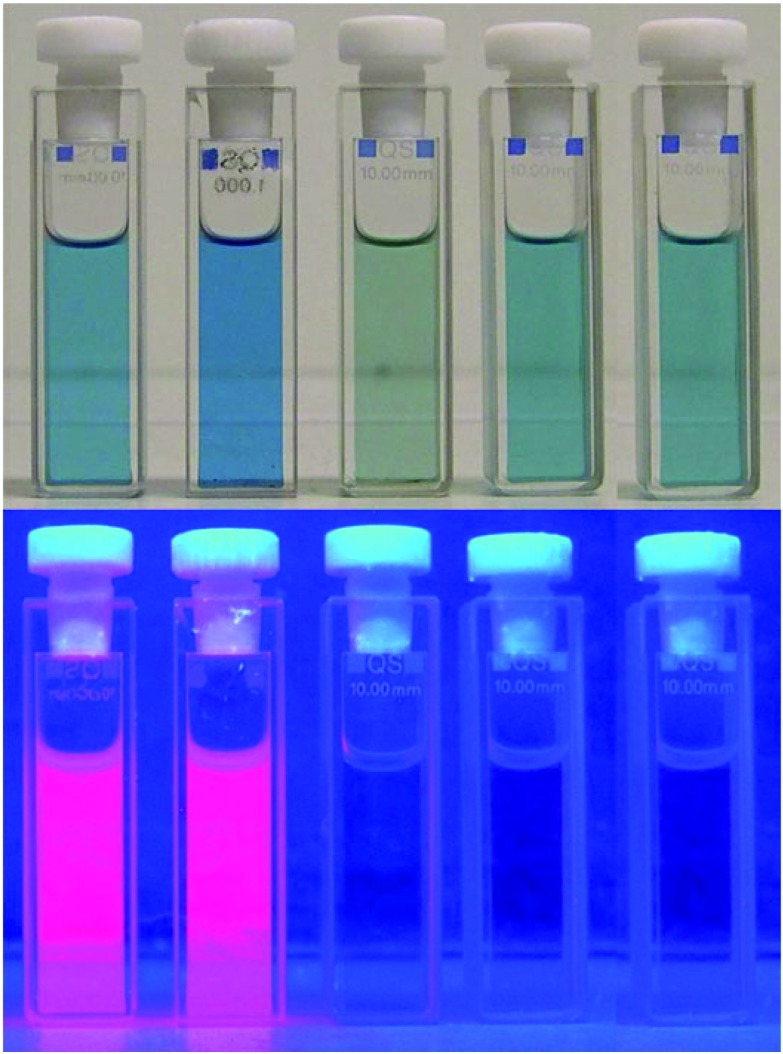
Solutions of metal complexes **M-3** (from left to right, with M = Zn(ii), Cd(ii), Ni(ii), Cu(ii) and Pd(ii)) of the PiCC **3** in MeOH, as observed under day light (top) or under UV-light (at 366 nm, bottom).

PiCC **3** is barely luminescent (weak emission near 615 nm), as are YCC **2** and most linear tetrapyrroles, which de-excite by rapid isomerization processes.^5^ Coordination of Zn-ions by PiCC **3** (gave the blue metal complex **Zn-3** and) lighted up an intense red luminescence (see Fig. 10): binding of Zn-ions and, likewise, of Cd-ions transformed the weakly luminescent PiCC **3** into the bright red fluorescent complexes **Zn-3** and **Cd-3**. Their emission around 650 nm was almost two orders of magnitude more intense than that of **3**. Appearance of such a strong luminescence by complex formation with closed-shell metal-ions can provide interesting insights and analytical applications with metal complexes **M-3**: as a consequence of the high affinity of **3** for transition metal-ions and high rates of binding to Zn(ii)- or to Cd(ii)-ions, analysis of the fluorescence of solutions of **3** allowed for the quantitative detection of Zn- and Cd-ions down to nM concentrations (*via* the luminescence of **Zn-3** or of **Cd-3**). A nearly linear correlation between the fluorescence intensity and the concentration of Zn(ii)- or of Cd(ii)-ions was observed at concentrations down to <10 nM, which was consistent with a 1 : 1 stoichiometry in the complexes. PiCC **3**, therefore, could serve as a reporter for Zn(ii)- and Cd(ii)-ions (and *vice versa*: Zn(ii)- and Cd(ii)-ions could be reporters for **3**), even at very low concentrations of the analytes.^26^ Thus, the fluorescence of their Zn- or Cd-complexes could be used to detect and track such PiCCs (*in vivo* or *ex vivo*) in plants.

Cu(ii)-ions displaced the Zn(ii)- and Cd(ii)-ions from the complexes **Zn-3** and **Cd-3**, which indicated the stronger binding capacity of **3** to Cu(ii). Ni(ii)- and Cu(ii)-complexes **Ni-3** and **Cu-3** showed negligible emission, as expected. Photo-excited **Pd-3** displayed fluorescence, with a maximum at 668 nm with low intensity (Fig. 10).

The main chromophore of the yellow catabolite YCC **2** (or its methyl ester **2-Me**) is the same as the one characteristic of bilirubin,^45^ and may, thus, also have similar capacities to bind metal-ions (see below). Indeed, our (preliminary) data suggest YCC **2** to have a significant affinity, *e.g.*, to Zn(ii)-ions.^72^ YCC **2** is only weakly luminescent. However, addition of Zn(ii)–acetate to a deoxygenated solution of **2** in DMSO resulted in the formation of the Zn-complex **Zn-2**, as indicated by a red shift of the UV/Vis-absorption maximum from 431 to 498 nm and by the appearance of a bright green fluorescence, with an emission maximum at 540 nm.^72^ In the luminescent **Zn-2** complex, the ligand is assumed to coordinate the Zn(ii)-ion in a bidentate form. In the presence of air and of an excess of Zn(OAc)_2_, solutions of **Zn-2** in methanol or in DMF undergo clean oxidation to the blue complex **Zn-3**.^26^ The presence of Zn(ii)-ions appears to accelerate oxidation of **2**, as similarly observed with BR.^73,74^ As the Zn(ii)-ion of **Zn-3** was easily removed by addition of phosphate (which precipitated Zn–phosphate), YCC **2** could be oxidized efficiently to PiCC **3** (*via* **Zn-2** and **Zn-3**).^26^

**Fig. 11:**
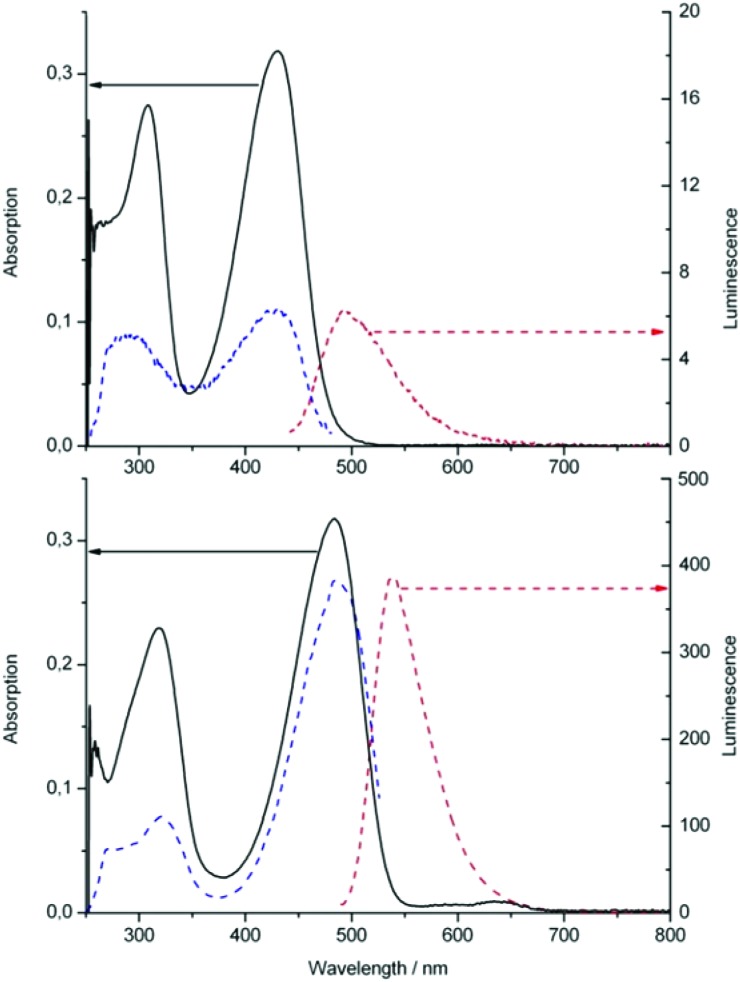
Absorption (full line, left scale), fluorescence (red broken line, right scale) and fluorescence excitation spectra (blue broken line, right scale) of solutions in DMSO of YCC methyl ester **2-Me** before (top) and after addition of Zn(OAc)_2_ (bottom). Complex formation with Zn(ii)-ions is indicated by the shift of the maxima of the absorption and of the intense emission (note different scales for luminescence, right).^72^

**Fig. 12:**
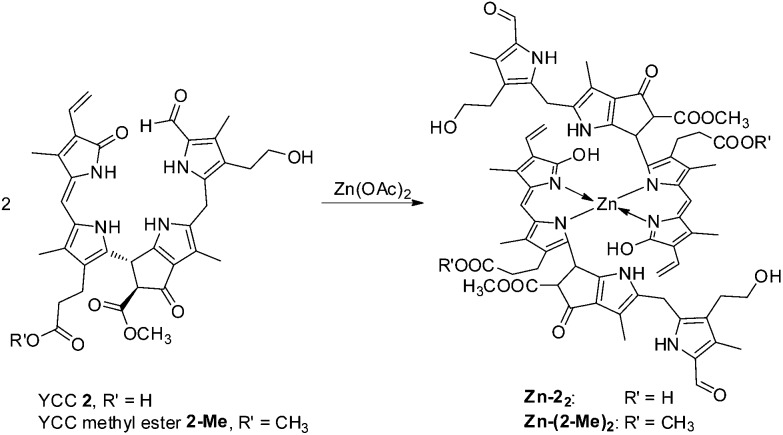
YCC **2** (and YCC methyl ester **2-Me**) and Zn(OAc)_2_ combine to the 2 : 1 complexes **Zn-2**_**2**_ (or **Zn-(2-Me)**_**2**_) in DMSO, in which the Zn(ii)-ions are presumably bound in a pseudo-tetrahedral, 4-coordinate fashion.^72^

Likewise, addition of an excess of Zn(OAc)_2_ to a deoxygenated (Ar-purged) solution of the methyl ester of YCC (**2-Me**) in DMSO led to the formation of a stable Zn(ii)-complex of **2-Me**, as indicated by a red shift of the absorption maximum from 430 to 484 nm, and by green luminescence with emission maximum at 538 nm (see Fig. 11). NMR-spectroscopic analysis in DMSO-d_6_ provided evidence for the structure of the 1 : 2-complex **Zn(2-Me)**_**2**_, in which the Zn(ii)-ions were encased in a pseudo-tetrahedral coordination mode by two molecules of **2-Me** that each acted as bidentate ligands. Upon binding of a Zn(ii)-ion to **2-Me** the signals of N23H and N24H disappeared in the ^1^H-NMR spectra, indicating coordination to N23 and N24. Furthermore, an apparent long range NOE-correlation between the non-coordinated ring B pyrrole–NH to the vinyl group in ring D (which is not observed in the NMR spectra of **2-Me**) is rationalized by an inter-ligand coupling between two coordinated molecules of **2-Me** (see Fig. 12).^72^ An ESI-MS analysis of isolated **Zn(2-Me)**_**2**_ supported the suggested 1 : 2 stoichiometry.

## Transition metal complexes of bilins

### Transition metal complexes of biliverdin

Biliverdin (BV) has been thoroughly investigated as ligand for transition metal ions, as reviewed recently.^24,25^ The structure of BV (as its dimethyl ester) was analyzed in the crystal, where it was found in a *Z*,*Z*,*Z*-configurated bis-lactam form with a weakly nonplanar helical conformation. Two neighbouring BV-dimethyl ester molecules were stitched together in the crystal in dimers by two lactam H-bonds.^75^ A non-natural bilindione, obtained from oxidation of tetra-mesophenyl-porphyrin (TPP), had similar structural characteristics.^76^

**Fig. 13:**
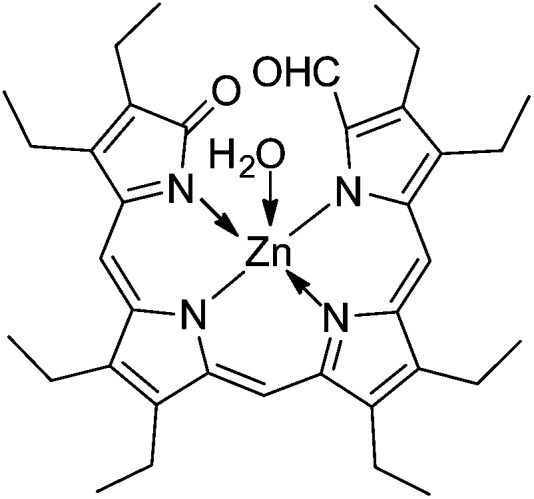
Formula of a model Zn(ii)-1-formylbiliverdinate, in which the Zn(ii)-ion is pentacoordinate due to ligation of a water molecule (structure derived from X-ray crystal analysis).

An early study reported a solution of *meso*-biliverdin (*m*BV) to change colour from blue to green upon addition of a solution of Zn(OAc)_2_ in MeOH under N_2_, due to formation of the Zn(ii)-complex of *m*BV.^77^ Analysis of the crystal structures of the Zn-complex of an ‘octaethyl-formylbiliverdinate’ (prepared from photo-oxidation of Zn-octaethylporphyrin) revealed the presence of a monomeric penta-coordinate Zn-complex as mono-hydrate (with four N and one axial H_2_O coordinating to Zn, see Fig. 13), as well as a dimer involving alternative bonding to two tetra-coordinate Zn-ions by two pairs of N-atoms from each ligand.^78^ A Zn-1,19-dideoxy-1,2,3,7,8,12,13,17,18,19-decamethylbiladiene-*a*,*c* featured a similar dimeric structure.^79^ In water or DMSO and in the absence of O_2_, binding of BV to Zn-, Cd- and Cu-ions was observed in a 1 : 1 stoichiometry. In aqueous solution, further oxidation reactions of various BV-metal complexes were observed.^24,74,80^

### Transition metal complexes of bilirubin

Bilirubin (BR) tends to be a more capricious ligand than BV, due to the ease of oxidation at flexible *meso*-position linking the two dipyrromethene groups. Crystalline BR displayed C4=C5 and C15=C16 bonds in a *Z* configuration, and a ridge tile structure of the whole tetrapyrrolic molecule, in which the two dipyrromethene groups were linked by the CH_2_ group.^6,24,66,81^ Various transition metal-ions, including Zn(ii), Cu(ii), Ni(ii), Co(ii), Fe(ii), Fe(iii), were tested for binding to BR or *meso*-bilirubin (*m*BR), as delineated in recent reviews.^24,25^ When Zn(ii)-, Cd(ii)- or Co(ii)-salts were added to a solution of BR in DMF or DMSO, fast colour changes to red were observed.^82^ A bathochromic shift of the band in the visible region (by approximately 80–100 nm) was observed, similar to the one seen when YCC **2** bound Zn(ii)-ions. A range of structures have been discussed for metal complexes of BR, while essential structural data were hardly obtained.^25,82^ Coordination of BR with Zn(ii)-, Cd(ii)- and Cu(ii)-ions was also studied in deoxygenated H_2_O, when formation of metal complexes was inefficient. Indeed, under O_2_, Zn- or Cd-complexes of the BR-oxidation product BV were obtained.^74^

## Zn-complexes of stercobilin and urobilins

Our knowledge is still scarce on metal complexes of partially reduced natural bilins, such as stercobilin (SB) and urobilin (UB).^24,25^ Titration of SB with Cu-ions gave UV/Vis-absorbance shifts that were interpreted by the formation of (a) Cu-SB complex(es). As with metal complexes of other bile pigments, treatment of Cu-complexes (*e.g.* of UB) with acid led to decomplexation.^83^

## Metal complexes of non-natural linear tetrapyrrole model compounds

**Fig. 14:**
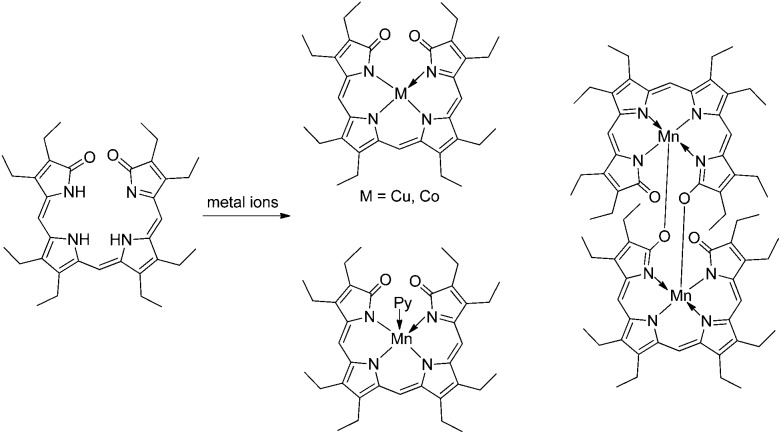
Octaethyl-biliverdinate model furnishes 4-coordinate Co- and Cu-complexes, and 5-coordinate Mn(iii)-complexes (either pyridine coordinated mono-nuclear or O-bridged di-nuclear complexes).^24^

Over a time of several decades, transition metal binding to a variety of non-natural bilins has been studied widely, as reviewed recently.^24,25^ Octaethylbilindione and the above-mentioned octaethylformyl-biliverdinate represented two easily accessible synthetic linear tetrapyrroles that were used as excellent models for BV, and binding, *e.g.*, Zn-, Co-, Ni- and Cu-ions as effectively tetra-coordinate ligands.^24,25,57,85,86^ Depending on the metal-ion, monomeric or dimeric metal complexes were observed in the crystals. Thus, in contrast, *e.g.*, to the complexes with Co- and Cu-ions, which were four-coordinate and monomeric, the five-coordinate Mn(iii)-ion gave a dimeric complex with octaethylbilindione in the crystal, where the lactam–O of one monomer-unit acted as the bridge to bind with the Mn(iii)-centre of the other moiety (see Fig. 14).^24,87^

**Fig. 15:**
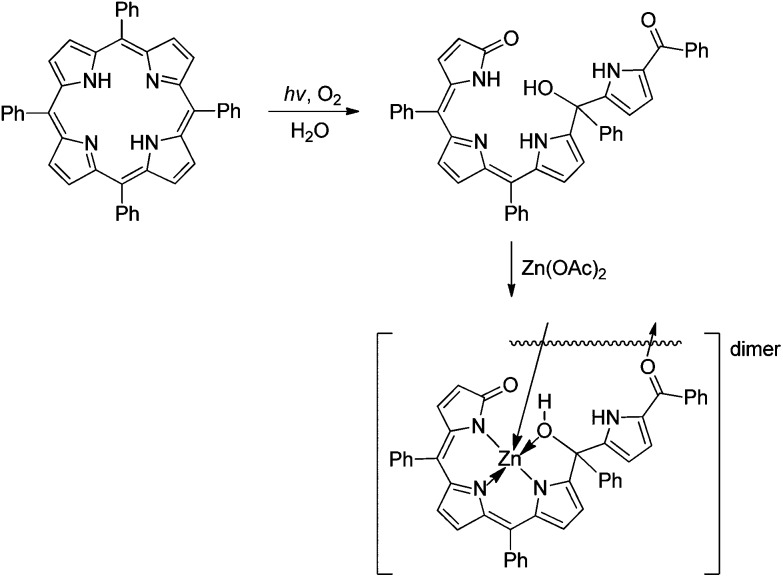
Violobilin-type products are obtained from photo-oxygenation of tetra-*meso*-phenylporphyrin in the presence of water, which furnish dimeric, O-bridged Zn(ii)-complexes, in which three N-atoms of the violobilin-type ligand coordinate the metal-ion (as deduced from a crystal structure analysis).^84^

An interesting, violet formyl-bilinone was isolated as main product of the photooxidation of *meso*-tetraphenylporphyrin (TPP) in H_2_O or MeOH, in which an OH or a MeO substituent was attached at one *meso*-position. This formyl-bilinone displayed a chromophore similar to the one of biliviolin, and of PiCC **3**, as well. Formation of a blue solution was observed, when the OH-derivative was treated with Zn(ii). On the basis of NMR data, the structure of the blue compound was proposed as the one of a biliviolin-type Zn complex.^59^ A crystal structure showed binding of three of the N-atoms and of the hydroxyl-group of the biliviolin-type ligand, confirming the proposed tridentate mode of N-coordination of the Zn(ii)-ion in a dimeric arrangement (Fig. 15).^84^

## Metal-binding capacities of phyllobilins relate to those of heme-derived bilins

As a rule, the highly unsaturated cyclic tetrapyrroles (porphyrins) act as tetra-coordinate ligands for transition metal ions. Indeed, the unsaturated linear derivative BV (a bilatriene obtained *via* heme-oxygenase) behaves in an analogous fashion.^24,25^ In contrast, typical ligands of the biliviolin-type provide only 3 N-atoms of the extended conjugated ligand chromophore for tridentate coordination to transition metal ions, keeping their isolated pyrrole unit de-coordinated.^59,84^ Interestingly, corresponding studies with the structurally related natural plant bilin phycoviolobilin appear to be unknown, so that the capacity for coordination of metal ions by the latter still remains to be established.

Among the phyllobilins, only PiCC **3** (a phyllobila-*b*,*c*-diene) has been studied extensively, so far, with respect to its capacity to bind transition metal ions.^26^ PiCC **3** is an effective tridentate ligand for biologically important transition metal ions. Free PiCC exhibits a remarkable ‘stretched’ structure (with *E*,*Z*-configuration at the C10=C11 and C15=C16 double bonds) that needs to isomerize to the *Z*,*Z*-form, in order be able to complex and wrap around a metal ion in a tri-coordinate fashion.^26^ Probably, the observed *E*-configuration in PiCC is due to steric effects associated with the substituted, ‘extra’ ring E of phyllobilins, which is a characteristic of these Chl-derived bilin-type linear tetrapyrroles that is attached to a pyrrole ring and the γ-*meso*-position (see, *e.g.* Fig. 7). The presence of ring E of the phyllobilins appears to be of lesser consequence in other respects, although it imposes a further geometric restriction and inhibits any *Z*/*E*-isomerisation around the C9–C10 bond, which also features partial double bond character. Similar to some synthetic biliviolin-type tetrapyrroles, the bila-*b*,*c*-diene PiCC **3** features a saturated, conformationally flexible 5-*meso*-position, which helps to avoid steric clashes between the 1- and 19-positions in the pseudo-cyclic structures of the ‘wrapped-up’ metal complexes.

The less unsaturated YCC **2** (a phyllobilene-c) exhibits a bidentate coordination pattern, as seen in its Zn-complex. So far, only Zn(ii)-complexes (**Zn-2**_**2**_ and **Zn-(2-Me)**_**2**_) have been studied,^72^ in which the coordination requirements of the Zn(ii)-ion are satisfied by binding two (bidentate) YCC-units, *i.e.* with YCC : Zn(ii) in 2 : 1 ratio. The conjugated system spanning rings C and D of YCC **2** occurs in a lactam form, which is indicated to undergo tautomerization to its lactim form in the neutral Zn-complexes **Zn-2**_**2**_ and **Zn-(2-Me)**_**2**_. The suggested coordination of a lactim form in **Zn-2**_**2**_ reminds of the proposed structure of the ‘formyl-biliverdine’ Zn-complex^78^ and of dipyrromethenes, which are strong chelators for metal ions.

**Fig. 16:**
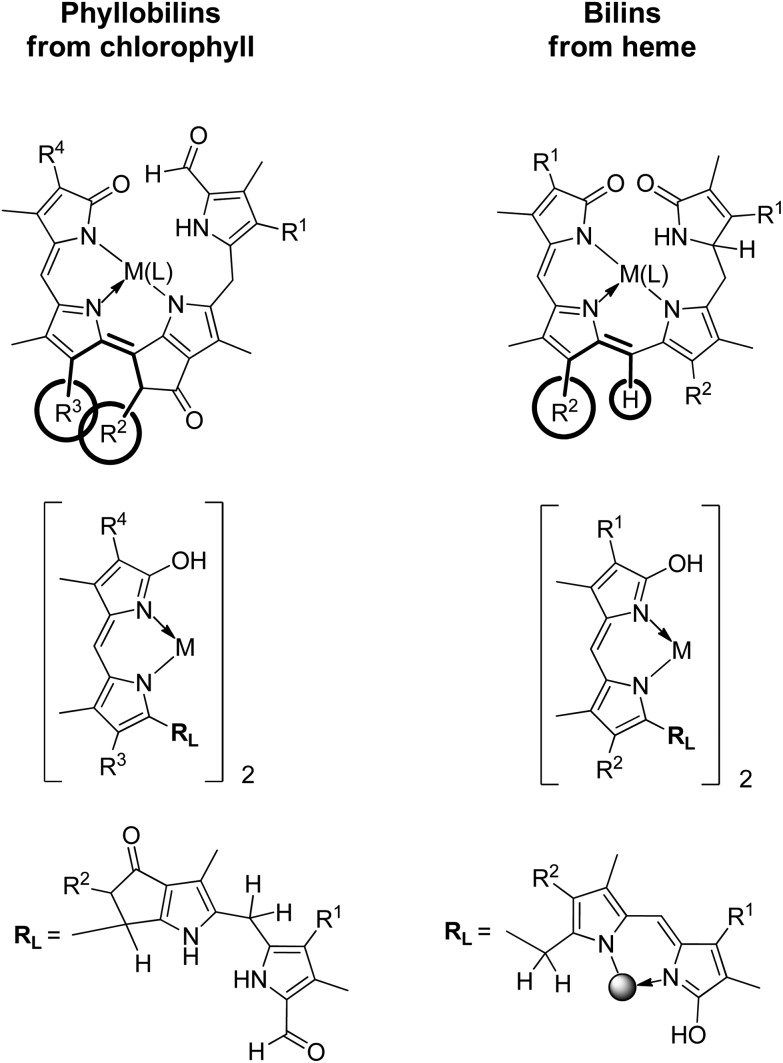
The coordination properties of phyllobilins and of corresponding bilins exhibit basic similarities. These are revealed here by a qualitative comparison of two representative types each, of PiCCs and YCCs (left) and of violobilins (VBs) and bilirubin (BR) (right). PiCCs and VBs provide a tridentate N-coordination pattern, YCCs and BR a bidentate N-coordination pattern (which is present twice in BR). Transition metal complexes of PiCCs require *Z*-configuration for the unsaturated bonds at the γ-*meso*-position (C10). This suggests a steric clash between the substituent R^2^ at the extra ring E and the propionic acid group (R^3^) at ring C. In metal complexes of heme-derived bilins a similar steric problem at the γ-*meso*-position would not exist.

Thus, the coordination properties of PiCCs and of YCCs relate to those of the heme-derived violobilins and BR (see Fig. 16). In this respect, it still remains to study the behaviour of the corresponding, partially unsaturated type-II phyllobilins (1,19-dioxobilin-type Chl-catabolites), which would have main chromophore structures corresponding to those of PiCCs and YCCs (1-formyl-19-oxobilins or type-I phyllobilins). Clearly, the two types of linear tetrapyrroles may have a roughly similar behaviour as ligands in complexes with transition metal ions, comparable to the behaviour of heme-derived dioxo-bilins and formyl-oxobilins.^24,25^

Interestingly, ‘non-fluorescent’ Chl-catabolites (NCCs) and dioxobilin-type NCCs (DNCCs), the two most abundant classes of the natural phyllobilins, are not expected to bind metal ions strongly, as they feature only un-conjugated pyrrolic rings. Likewise, natural bilane-type tetrapyrroles (which occur in the course of the biosynthesis of the porphyrinoids) are not known to bind transition metal ions. In contrast, ‘fluorescent’ Chl-catabolites (FCCs), and the more unsaturated red Chl-catabolites (RCCs), exhibit structures that suggest a capacity for effective metal ion coordination.

## Outlook

In the course of the last 25 years, the highly abundant catabolites of Chl, named ‘phyllobilins’, were discovered and explored as a new type of natural linear tetrapyrroles.^9^ Most of the original chemical work in this area centred on structure elucidation of the growing class of the phyllobilins. It revealed the biological importance of mainly colourless chlorophyll-catabolites that are hardly able to coordinate transition metal ions. However, in addition, partially unsaturated, coloured bilin-type chlorophyll catabolites were discovered in the course of this work. As reviewed here, these may display a capacity to complex metal ions comparable to, or even superior to that of well-investigated heme-catabolites, such as biliverdine (BV) or bilirubin (BR).

Among the phyllobilins examined with respect to binding of transition metal ions, the pink-coloured phyllobiladienes, called PiCCs, have been most thoroughly studied. Compared to BV or to BR, which may bind a transition metal ion in a tetra-dentate or (twice) bidentate fashion, PiCCs are effective tridentate ligands. When coordinating transition metal-ions that prefer to be tetra-coordinate, the PiCC ligand thus leaves one coordination site unoccupied. This ‘vacancy’ may be used for coordination by an external ‘fourth’ ligand. This feature offers an opportunity for attaching PiCC metal complexes to correspondingly dispositioned bio(macro)molecules, *e.g.*, to proteins or to nucleobases, providing PiCC metal complexes with potentially interesting biological functions and applications. At the same time, coordination of closed-shell metal ions to the barely luminescent PiCCs, such as Zn(ii)- or Cd(ii)-ions, induce such phyllobilin metal complexes to exhibit bright fluorescence. Binding of Zn(ii)- or Cd(ii)-ions to PiCC may occur in plants, where the strong luminescence could be used as diagnostic optical effects to detect these complexes *in vivo*.

Aside of the studies with PiCCs, the capacity of phyllobilins as ligands for transition metal ions has barely been investigated. As indicated here briefly, yellow chlorophyll catabolites (such as YCC **2** and its methyl ester **2-Me**) are able to coordinate Zn(ii)-ions and give green luminescent complexes. However, the presence of metal-ions and metal-chelation may enhance decomposition, or oxidation processes of phyllobilins, as seen with the oxygen sensitive YCC.

There are isolated reports on the natural occurrence of transition metal complexes of heme-derived bilins, and on presumed biological roles of their transition metal complexes.^24,68^ Thus, a Zn-complex of BV was identified as pigment in the eggshells of birds.^24^ A Cu(ii)-complex of BR appears to cut DNA in the presence of molecular oxygen,^88^ a feature shared by the Cu-complex of tripyrrolic alkaloids, named prodigiosins.^71^ Phyllobilins may be expected nowadays to have biological roles, as well, which are, however, still entirely elusive. Phyllobilins are linear tetrapyrroles that do represent an interesting new group of multi-dentate ligands for biologically important transition metal ions. In analogy to bilins, transition metal complexes of coloured phyllobilins have properties that may be physiologically relevant and beneficial *e.g.* in plants, as sensitizer for singlet oxygen,^89,90^ act as additional toxins against pathogens,^71^ or play a part in heavy metal transport and detoxification.^91^ Clearly, in that respect, only the ‘top of the iceberg’ has been uncovered by our studies, so far, and phyllobilins and their transition metal complexes are expected to remain the topic of further interesting discoveries.
